# Surface-phonon-polariton-enhanced photoinduced dipole force for nanoscale infrared imaging

**DOI:** 10.1093/nsr/nwae101

**Published:** 2024-03-18

**Authors:** Jian Li, Junghoon Jahng, Xuezhi Ma, Jing Liang, Xue Zhang, Qianhao Min, Xiao-Liang Wang, Shuangjun Chen, Eun Seong Lee, Xing-Hua Xia

**Affiliations:** State Key Lab of Analytical Chemistry for Life Science, School of Chemistry and Chemical Engineering, Nanjing University, Nanjing 210023, China; Hyperspectral Nano-Imaging Team, Korea Research Institute of Standards and Science, Daejeon 34113, South Korea; Institute of Materials Research and Engineering, Agency for Science, Technology and Research, Singapore 138634, Singapore; State Key Lab of Analytical Chemistry for Life Science, School of Chemistry and Chemical Engineering, Nanjing University, Nanjing 210023, China; State Key Lab of Analytical Chemistry for Life Science, School of Chemistry and Chemical Engineering, Nanjing University, Nanjing 210023, China; State Key Lab of Analytical Chemistry for Life Science, School of Chemistry and Chemical Engineering, Nanjing University, Nanjing 210023, China; State Key Lab of Analytical Chemistry for Life Science, School of Chemistry and Chemical Engineering, Nanjing University, Nanjing 210023, China; College of Materials Science and Engineering, Nanjing Tech University, Nanjing 210009, China; Hyperspectral Nano-Imaging Team, Korea Research Institute of Standards and Science, Daejeon 34113, South Korea; State Key Lab of Analytical Chemistry for Life Science, School of Chemistry and Chemical Engineering, Nanjing University, Nanjing 210023, China

**Keywords:** surface phonon polariton, photoinduced dipole force, nanoscale infrared imaging, ultrathin sample

## Abstract

The photoinduced dipole force (PiDF) is an attractive force arising from the Coulombic interaction between the light-induced dipoles on the illuminated tip and the sample. It shows extreme sample-tip distance and refractive index dependence, which is promising for nanoscale infrared (IR) imaging of ultrathin samples. However, the existence of PiDF in the mid-IR region has not been experimentally demonstrated due to the coexistence of photoinduced thermal force (PiTF), typically one to two orders of magnitude higher than PiDF. In this study, we demonstrate that, with the assistance of surface phonon polaritons, the PiDF of c-quartz can be enhanced to surpass its PiTF, enabling a clear observation of PiDF spectra reflecting the properties of the real part of permittivity. Leveraging the detection of the PiDF of phonon polaritonic substrate, we propose a strategy to enhance the sensitivity and contrast of photoinduced force responses in transmission images, facilitating the precise differentiation of the heterogeneous distribution of ultrathin samples.

## Teaser

Infrared photo-induced dipole force enhanced by substrate surface phonon polariton has been used for high-contrast imaging of ultrathin samples.

## INTRODUCTION

Force-based nanoscale infrared (nano-IR) imaging techniques, including photothermally induced resonance (PTIR) [1], peak force infrared (PFIR) [2] and photoinduced force microscopy (PiFM) [3,4], offer the capability to investigate samples at the nanoscale by directly imaging their intrinsic vibrational modes—the ‘fingerprint’ identities of materials. These techniques are crucial tools for addressing questions in nanophotonics, polymer science and biology [5–14]. The primary signals in force-based nano-IR arise from the photothermal expansion of the sample beneath an illuminated atomic force microscopy (AFM) tip, locally modulating the detected force and contributing to the nano-IR contrast.

Depending on the applied detection technique, the photoinduced thermal force (PiTF) can be either a repulsive force (PTIR, PFIR) or an attractive force (PiFM), directly proportional to the absorption coefficient of a sample, establishing a strong correlation between nano-IR spectra and far-field IR spectra. In addition to the IR absorption coefficient, PiTF is influenced by the thermal properties and thickness of samples, where the latter correlates directly with the morphology of the sample. Consequently, imaging ultrathin samples with PiTF becomes challenging and necessitates specific signal enhancement strategies due to the reduced sampling volume, limiting its application in distinguishing detailed heterogeneous distributions inside and between ultrathin samples.

**Figure 1.**
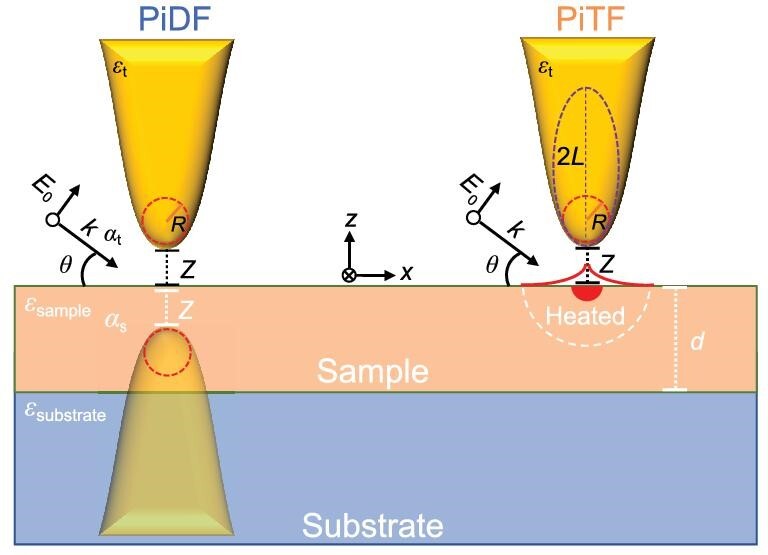
Schematics of photoinduced thermal force (PiTF) and photoinduced dipole force (PiDF). The tip is modeled as an ellipsoid with a length of 2*L*. The plane wave light is illuminated to the sample with an angle of *θ. z* is the gap distance from the tip to the sample surface and *d* is the thickness of the sample.

PiFM operates in the non-contact region so that the thermal expansion underneath the tip is mediated by the force gradient between the tip and the sample, and eventually manifests as the attractive forces [15]. In addition to that, when the light illuminates the tip-sample junction, a photoinduced dipole force (PiDF), arising from the Coulombic interaction between the light-induced dipoles on the tip and the sample (Fig. 1), is predicted to contribute to the measured PiF [16–18], which is also described in Supplementary Sections 3 and 4 (Figs S1–S4). This force reflects the properties of the real part of the permittivities of the tip and the sample, which can show a dispersive spectral line shape, akin to the amplitude of scattering-type scanning near-field optical microscopy (s-SNOM) [19–21]. Indeed, as illustrated in Fig. 1, when the tip is fabricated with an electrically conductive material, the incident light as an electromagnetic field can polarize the tip-apex, effectively establishing an electric dipole. Then, this induced tip dipole can generate an image dipole within a substrate surface in close proximity to the tip, which leads to an extremely localized electric dipole–dipole interaction force, i.e. the dipole force, on the tip [22]. Additionally, by incorporating a resonant nanostructure and employing structured light illumination, the tip can also be polarized as an exclusive magnetic dipole, enabling the direct detection of the magnetic dipole force [23]. Therefore, the PiDF is expected to show significantly localized multidimensional electromagnetic responses compared to those of the PiTF, so that imaging with PiDF could yield information far beyond the scope of PiTF, such as sample-tip distance, localized refractive index differences, and various types of subtle electric and magnetic near-field properties. However, in the mid-IR region, the PiDF is typically one to two orders of magnitude smaller than the PiTF in most polymers (positive real part of permittivity) so that it is very challenging to demonstrate experimentally [24,25].

Surface phonon polaritons (SPhPs) are collective surface modes that hybridize photons with lattice vibrations in polar insulators, providing a unique means for nanoscale control of light–matter interactions. Since the photoinduced dipoles under the tip-sample geometry can be strongly enhanced due to this surface mode at the negative real part of the permittivity, certain metals or polar crystals can exhibit a strongly enhanced dipole response [26,27]. In contrast to the confined field on metals due to surface plasmon polaritons [28], which have relatively high losses, the surface phonon polaritons on polar crystals such as c-quartz, SiC and calcite exhibit relatively lower losses, leading to much stronger field enhancement between the tip and the sample [26,29]. Thus, introducing this surface mode provides a unique means to enhance the PiDF [30]. However, the PiTF, due to this strong tip-enhanced near-field, is also expected to be enhanced [24]. Previous PiFM investigations have had great success in detecting phonon polariton modes in the IR range, yet the force contribution to the signal has not been clearly investigated [31,32]. One of the ideas to overcome this limit is using materials that exhibit negligible absorption in the mid-IR to set up the plasmonic structure [33]. The researchers successfully demonstrated the dispersive line shape of the epsilon near zero (ENZ) mode of InAsSb/GaSb semiconductor grating in the mid-IR range, which well corresponds to the s-SNOM amplitude spectrum.

In this study, we show that the surface phonon polaritons of quartz can enhance its PiDF to surpass its PiTF. Having successfully detected PiDF, we devised a nanoscale contrast IR imaging strategy capable of finely distinguishing the heterogeneous distribution of ultrathin samples.

## RESULTS AND DISCUSSION

### Investigation of PiDF on quartz

In principle, the PiDF can be qualitatively elucidated by employing a straightforward point dipole model [3,16,17]. This model (schematically shown in Fig. 1) involves the effective polarizabilities of both the AFM tip (*α_s_*) and the sample (*α_t_*), which can be expressed through the near-field (quasi-electrostatic) reflection coefficient (*β*), defined as *α_s_ = βα_t_* in an image dipole theory. On the other hand, the PiTF is explicable through the assessment of thermal expansion and the gradient of the tip-sample interaction force, representing the van der Waals force in the non-contact region. The expressions for these forces are provided as follows:


(1) \begin{eqnarray*}
{{F}_{{\mathrm{dip}}}} \propto - \frac{{Re\left\{ {\beta \left( \\lesssimmbda \right)} \right\}}}{{{{z}^4}}}{{\left| {{{{\mathrm{\alpha }}}_{\mathrm{t}}}{{E}_0}} \right|}^2} + {{F}_{{\mathrm{sc}}}} \quad \quad \ \ \ (z > {{r}_0})
\end{eqnarray*}

(2) \begin{eqnarray*}
{{F}_{{\mathrm{th}}}} \propto - \frac{{\partial {{F}_{{\mathrm{ts}}}}}}{{\partial z}}\sigma {{l}_z}{{\tau }_{{\mathrm{th}}}}\int {{a}_{{\mathrm{abs}}}}\left( \\lesssimmbda \right)|{{E}_{{\mathrm{tot}}}}{{|}^2}{\mathrm{d}}{{V}_{{\mathrm{abs}}}}\quad (z > {{r}_0})
\end{eqnarray*}

where *r*_0_, *R, E*_0_ and *z* are the interatomic distance (∼0.3 nm), tip radius, incident electric field and instantaneous tip-sample distance from the tip end to the surface. *F*_sc_, *F*_ts_, *σ, l_z_, τ*_th_, *a*_abs_ and *E*_tot_ are the scattering force due to the incident light momentum [16], the tip-sample interatomic force, thermal expansion coefficient, heating length of the sample, effective thermalization time, effective absorption coefficient and total electric field inside the sample.

Equation (1) is a highly simplified equation that is only valid when the distance between the tip and the sample is notably larger than the size of the tip [16,17]. Although the quantification of this force involves further considerations of the tip geometry, this point dipole model still adequately captures the physics at the tip-sample junction under light illumination and helps to intuitively understand the spectroscopic information. Since *β* = *(ε−*1*)/(ε+*1) with the complex permittivity (*ε = ε′ + iε′′*) of an isotropic material, a maximum PiDF can be expected at *ε′* = −1 and *ε′′ =* 0, which is the resonance condition of the image dipole under spherical tip-planar sample geometry, so called tip-induced near-field resonance (tNR) or image dipole resonance (IDR) [26–28,34]. The physical meaning of this resonance condition is closely related to the surface mode. Since the resonance also depends on the tip-sample geometry, such as gap distance and tip shape, the amplitude and frequency can be more accurately predicted by implementing the finite dipole model with the elongated tip geometry [35–37], also shown in Supplementary Section 4.

The total thermal expansions underneath the tip can be considered as the summation of the near-field-based tip-enhanced thermal expansion (Δ*L*_t_) and the incident far-field-based global thermal expansion (Δ*L*_g_), whose heating volumes are different [15,38], and are derived in Supplementary Section 3. In Equation (2), the description without $\frac{{\partial {{F}_{{\mathrm{ts}}}}}}{{\partial z}}$ is the total thermal expansion given as ${\mathrm{\Delta }}{{L}_{{\mathrm{tot}}}} = {\mathrm{\Delta }}{{L}_{\mathrm{t}}}( z ) + {\mathrm{\Delta }}{{L}_{\mathrm{g}}}$.

**Figure 2.**
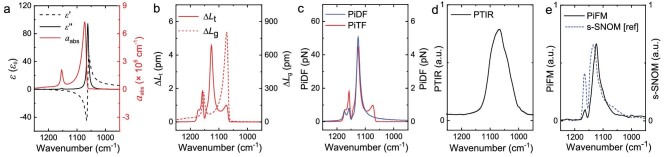
PiFs of quartz. (a) Real (dashed black line) and imaginary (solid black line) parts of permittivity of quartz from library data [35] and the calculated effective absorption coefficient (red solid line). (b) Tip-enhanced (red solid line) and global (red dashed line) thermal expansions, analytically calculated by implementing the finite dipole method. (c) PiDF (blue solid line) and PiTF (red solid line) for the surface response, analytically calculated by implementing the finite dipole method. (d) Measured resonance-enhanced contact mode PTIR (AFM-IR) spectrum on pure quartz. (e) The PiFM spectrum, measured by a driving amplitude of 1 nm with the 90% setpoint of the amplitude on pure quartz, and the previously measured s-SNOM amplitude spectrum on pure quartz (blue dashed line) [40]. All the calculation parameters are given in the supplementary data.

Quartz, a well-known polar crystal, is chosen as the substrate in this study. Its transverse optical phonon (TO) resonance is near 1075 cm^−1^ (Si–O–Si stretching mode), the real part of permittivity (*ε′*) is negative, ranging from 1070 to 1200 cm^−1^ and the imaginary part (*ε′′*) is exceptionally low near the longitudinal optical (LO) frequency, as shown in Fig. 2a. Note that the complex permittivity of quartz is revisited from the previous measurement [35] and red-shifted by 10 cm^−1^ to fit our results. According to ref. [39], the effective absorption coefficient can be calculated using the equation of ${{a}_{{\mathrm{abs}}}}( \\lesssimmbda ) \approx \frac{{4{\mathrm{\pi }}}}{\\lesssimmbda }\frac{{9{\mathrm{Re}}[ n ]{\mathrm{Im}}[ n ]}}{{{{{({\mathrm{Re}}{{{[ n ]}}^2} + 2)}}^2}}}$, where *n* is the complex refractive index, whose resonance slightly shifts (Fig. 2a) to the resonance of *ε′′* due to the large negative *ε′* (for detailed calculations, please refer to Supplementary Section 3).

The calculated Δ*L*_t_ and Δ*L*_g_ of the quartz are plotted in Fig. 2b as solid and dashed red lines, respectively. The global thermal expansion directly follows the absorption coefficient, since it only considers the incident light absorption by the sample without the tip, which shows the Si–O–Si vibrational absorption resonances of the quartz at 1075 and 1154 cm^−1^. On the other hand, for the tip-enhanced thermal expansion, it shows the maximum peak near 1125 cm^−1^, where there is the tip-induced near-field resonance as well as the Si–O–Si vibrational absorption resonances.

In the heterodyne PiFM operation, the far-field-based response, such as the scattering force and the global thermal expansion force, can be successfully rejected with a small oscillation amplitude [38]. Hence, the surface response of PiDF and PiTF induced by the tip-enhanced near-field are considered in the heterodyne PiFM measurement, which is calculated in Fig. 2c. As expected, the strongly enhanced dipole force is shown near the tip-induced near-field resonance (1125 cm^−1^). The magnitude of PiDF is typically one order higher than that of the PiTF under the same tip-enhanced field with the deviated spectrum. Since the fused quartz has an extraordinarily small thermal expansion coefficient (0.55 × 10^−6^/K), which is two orders smaller than those of typical polymers, the Δ*L*_t_ of fused quartz is negligibly small by a few picometers even under the strong phonon polaritonic field. In other words, even if the surface of quartz can be heated up by the tip, it rarely expands because its heating volume is very small. Moreover, the origin of the tNR is attributed to the strong coupling between the surface phonon polariton of quartz and the near-field from the tip. This coupling acts as a strong oscillator with a negative permittivity, resulting in a narrow spectral line width. In contrast, the broad PTIR spectral feature associated with Si–O–Si resonance is directly influenced by the far-field absorption without coupling to the tip, reflecting a weak oscillator which has a positive permittivity, and resulting in the broad phonon vibrational spectral line width of quartz. This is also demonstrated by using the resonance-enhanced PTIR measurement, which probes the total thermal expansion of the quartz. In Fig. 2d, the PTIR spectrum clearly shows that the tip-enhanced thermal expansion under this phonon polaritonic field is negligible by showing no peak at 1125 cm^−1^, but showing the broad peak at 1075 cm^−1^, ascribed to Si–O–Si vibrational absorption.

We obtain the PiFM spectrum of pure quartz by using PiFM heterodyne modes with a 1 nm small oscillation amplitude to extract the near-field behavior in Fig. 2e. The PiFM spectrum of pure quartz clearly reveals the tip-induced near-field resonance at 1125 cm^−1^, which well corresponds to the expected PiDF spectrum (blue solid line) in Fig. 2c. The spectral shape very closely resembles the amplitude of the s-SNOM result (blue dashed line in Fig. 2e) from ref. [40], which directly follows the real part of the effective polarizability of quartz. Hence, quartz is a perfect material for demonstrating the enhanced dipole force response by reducing the thermal expansion behavior.

**Figure 3.**
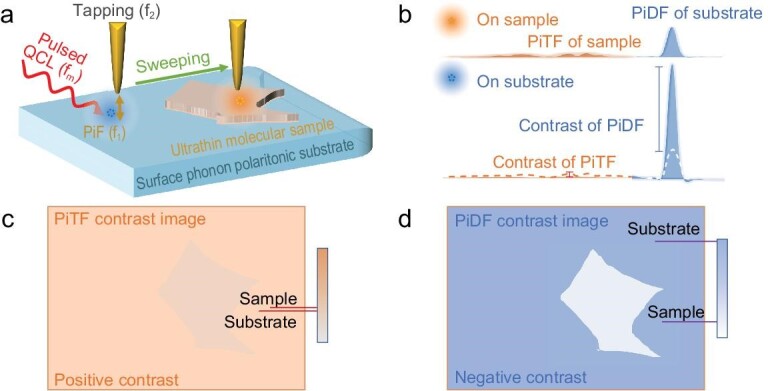
Phonon-polariton-enhanced nano-IR contrast imaging platform. (a) Sketch of the substrate-enhanced nano-IR contrast imaging platform based on a polar crystal substrate under the metallic tip. (b) Typical PiFM spectra were observed on the sample near its IR resonance and on the substrate near the tip-induced near-field resonance. (c, d) Schematics for the imaging of (c) the PiTF of the sample, and (d) the PiDF of the substrate, and a layered sample deposited on a PiDF-dominant substrate, as depicted in (a).

In the context of quartz, the PiDF demonstrates a more pronounced relationship with the tip-quartz distance (∼*z*^−4^) than the PiTF, which typically exhibits a relationship of ∼*z*^−3^. This prompts the proposal of a general approach for nano-IR contrast imaging of ultrathin samples, as outlined in Fig. 3a, where an ultrathin sample resides on top of quartz. The anticipated PiFM spectrum for a typical ultrathin sample, characterized by a positive real part of the permittivity (weak oscillator), is expected to manifest weak PiTF and PiDF near its infrared (IR) resonance [3,25]. However, a significant PiDF change is anticipated near the tip-induced near-field resonance of quartz substrate (Fig. 3b). These spectral distinctions contribute to the contrasts in nano-IR imaging. Notably, the PiDF response on quartz exhibits a more conspicuous signal variation with respect to sample thickness compared to the PiTF of the sample. For ultrathin samples, PiDF imaging on quartz presents an opposite contrast with enhanced sensitivity compared to the nano-IR contrast imaging with the PiTF of the sample (Fig. 3c and d).

### Principle demonstration of enhanced nano-IR contrast imaging

**Figure 4.**
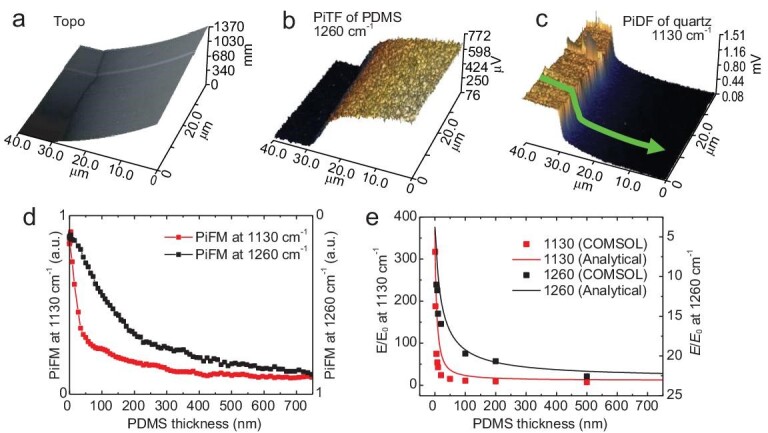
Nano-IR contrast imaging of PDMS on quartz. (a) AFM image of a PDMS wedge on the quartz surface. (b, c) PiFM images were recorded at 1260 and 1130 cm^−1^ within the same region, respectively. (d) Comparison of the line cuts of PiFM signals at 1260 and 1130 cm^−1^ along the green arrow in (c). The scale of the PiFM signal at 1260 cm^−1^ is inverted to directly compare with the one at 1130 cm^−1^. (e) COMSOL (dots) and analytical calculation (solid lines) of near-fields at the tip end with respect to the PDMS thickness at 1260 and 1130 cm^−1^. The calculation parameters can be found in Supplementary Section 5.

A polydimethylsiloxane (PDMS) wedge was prepared on a quartz substrate to demonstrate substrate-enhanced nano-IR contrast imaging. The near-field responses of PDMS thickness were tracked by implementing a small oscillation amplitude in a heterodyne PiFM measurement. The thickness of the PDMS layer was measured with AFM and found to increase from 0 to 1370 nm (Fig. 4a). According to the relative permittivity of PDMS and quartz (Fig. S5), PDMS shows absorption at ∼1260 cm^−1^, which can be distinct from the PiDF of quartz, and the PiFM images for PDMS and quartz were collected (Fig. 4b and c). To get comprehensive insights into the correlation between the PiFM signal and PDMS thickness, we extracted these data along the green line in Fig. 4c and replotted them in Fig. 4d. The scale of a signal at 1260 cm^−1^ is inverted to directly compare with the PiFM signal at 1130 cm^−1^. The PiFM signal at 1260 cm^−1^ quickly increases from 0 to 200 nm in thickness, and then gradually reaches a plateau as a function of PDMS thickness, which corresponds to the tip-enhanced thermal expansion behavior on a low refractive index substrate. In addition, the PiFM signal at 1130 cm^−1^ drastically decreases to 25% when the PDMS thickness reaches 75 nm, and then gradually reaches a plateau independent of the PDMS thickness.

The observed opposite phenomena can be explained as follows: at 1260 cm^−1^, the absorption of quartz is nearly zero (black dashed line), and therefore, quartz acts as a transparent IR material with the Fresnel's reflection coefficient. Since the field enhancement at the tip-end depends on the real part of the permittivity of the sample, it increases and then gets saturated with the increase of PDMS thickness, where *ε′*_PDMS_ ≈ 1.88 > *ε′*_quartz_ ≈ 0.55 at 1260 cm^−1^, as illustrated in Fig. S5. This is demonstrated by the analytical calculations (black solid line) and COMSOL simulations (black dots) in Fig. 4e. However, at 1130 cm^−1^, the real part of permittivity of quartz approaches the tip-induced near-field resonance, while the PDMS has a finite positive value (*ε′*_PDMS_ ≈ 1) as shown in Fig. S5, respectively. In this case, the field on quartz is strongly enhanced due to the amplified surface mode in the nanocavity geometry between the metallic tip and quartz substrate, while PDMS is regarded as the IR spacer (with no reflection) with a mediocre absorption. Thus, a very rapid decay of the PiFM signal is observed as the PDMS thickness increases, which is in good agreement with the simulations in Fig. 4e (a more detailed discussion is described in Supplementary Section 6, Fig. S6). These results provide clear evidence that the PiDF can be employed for sensitive nano-IR imaging of ultrathin samples under nanocavity geometry with improved contrast and sensitivity.

### High-contrast IR imaging of layered COFs

**Figure 5.**
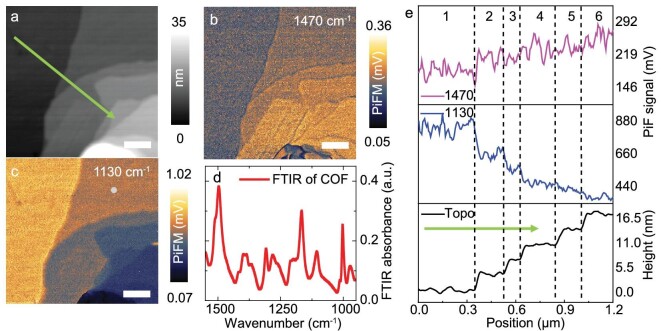
(a) AFM image of layered COFs on c-quartz surface, scale bar: 200 nm. The green arrow indicates the direction for signal extraction in (e). (b, c) PiF images of the same region recorded at 1470 cm^−1^ (PiTF of COF is dominant) and 1130 cm^−1^ (PiDF of quartz is dominant), scale bar: 200 nm. (d) PiF spectra collected on the different layers of the COFs layers. (e) Signals along the green arrow of topography and PiF images as indicated in (a).

In this demonstration, we image a fragment of layered covalent organic frameworks (COFs). According to the Fourier-transform infrared spectroscopy (FTIR) (Fig. 5d) and PiF (Fig. S7) spectra of COFs, the COFs have an IR absorption peak near 1490 cm^−1^, attributed to the C–H deformation vibrations of methyl groups. Since the FTIR spectrum shows minimum absorption near 1130 cm^−1^, it is expected that the PiDF of c-quartz is dominant to the PiTF of COFs. Consequently, nanoscale IR imaging of COFs can be achieved using both the PiTF of the COFs and the PiDF of c-quartz. Further exploration involves studying the PiF responses of COFs within a confined area of 1 μm × 1 μm. Fig. 5a presents the AFM image of the selected area, displaying layered structure. Simultaneously, PiF images are recorded at different wavenumbers along with the AFM images (Fig. 5b and c), indicating that utilizing the PiTF of the COFs and PiDF of c-quartz can distinguish the various layers of COFs. As anticipated, the signal contrast of the PiF image recorded at each wavenumber is conspicuously opposite. With increasing thickness, a lower PiDF of c-quartz and higher PiTF of the COFs are observed. Additionally, distinct geometric features such as sharp corners are distinguishable in the PiF images.

The line profiles of height and PiF signals along the green arrow indicated in the topography are plotted in Fig. 5e. The line profile of topography exhibits six distinct layers within 16.5 nm height variations, which can also be well distinguished from the AFM images that are numbered according to the gray scale. Normalized PiF signals along the same line are plotted in different panels. The maximum and minimum values of each panel are set as 150% and 50% of the average value of maximum and minimum PiF signals in each line. Larger signal variations are observed on the line profile at 1130 cm^−1^ (PiDF of quartz is dominant) than at 1470 cm^−1^ (PiTF of COF is dominant), especially at the initial layers. This is further confirmed by the data analysis shown in Table S2. Here, Δ|I_PiF_| is defined as the division of the highest PiF signal observed with PiF signal strength difference between different layers. At the first stage, with the thickness of COFs layers increasing to 4.2 nm, the c-quartz PiDF-dominant signal decreases by 24.8% at 1130 cm^−1^, while the COF PiTF-dominant signal increases by only 7.0% at 1470 cm^−1^. The > 3-fold increase in sensitivity suggests that the PiF signal of substrate phonon polariton could be more sensitive than the molecular signal.

### Resolving subsurface defects

**Figure 6.**
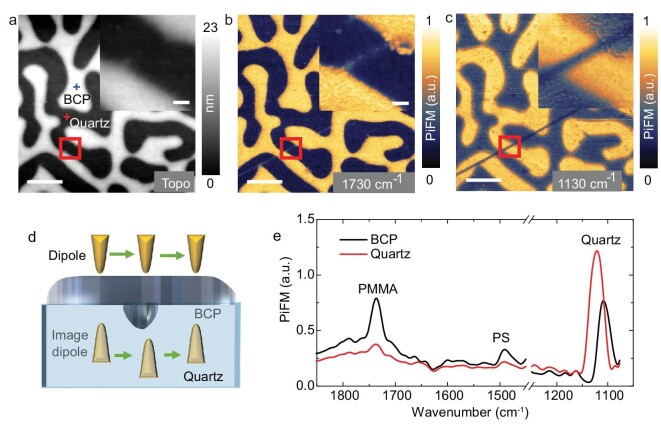
Subsurface imaging of deposited block copolymer clusters. (a) Topography of a clustered PS-*b*-PMMA on a quartz surface. Scale bar: 1 μm. PiFM images recorded at (b) 1730 cm^−1^ and (c) 1130 cm^−1^ in the same region shown in (a). The insets show the zoomed-in area corresponding to the red rectangle, with 256 × 256 resolution. (d) Diagnosed nanostructure with nano-IR contrast images. (e) Point PiFM spectra on BCP (blue cross on topography) and quartz substrate (red cross on topography).

For deposited nanodevices, subsurface defects could affect device performance. The established technique of substrate-enhanced nano-IR contrast imaging might be applied for the subsurface diagnosis of the deposited nanodevices. To demonstrate this, we have used quartz as a substrate covered with a block copolymer (BCP). The clustered BCP film with a thickness of ∼16 nm, consisting of PS-b-PMMA (21k–10k; PS: polystyrene, PMMA: polymethyl methacrylate), is shown in Fig. 6a. The PiFM spectrum of the BCP film (at the blue cross in Fig. 6a), revealing two peaks at 1730 cm^–1^ (carbonyl C=O vibrations of PMMA) and 1492 cm^–1^ (C=C bonds of PS), indicates the existence of both PMMA and PS components in the cluster. The tip-induced near-field resonance peak is evident at 1125 cm^–1^, depicted by the solid red line. Additionally, a heightened response near 1100 cm^–1^ is observed in the molecular resonance of PMMA (solid black line), attributed to its interaction with the surface phonon polariton field of the substrate, resulting in a redshift of the peak. This effect, previously reported, also helps to probe the molecular response in nanospectroscopy [27,40].

The PiFM image in Fig. 6b was recorded at 1730 cm^–1^ in the same region depicted in Fig. 6a. Although the nanoscale structures of BCP can be observed in the zoomed-in image of the red rectangular area in the inset of Fig. 6b, the thin line of PMMA is barely visible in the inset of the topography and PiTF images in Fig. 6a and b, respectively. On the other hand, the contrast image at 1130 cm^–1^ exhibits a remarkable feature, as shown in Fig. 6c, with a bright line, which is barely discernible in the topography and PiFM images at 1730 cm^–1^. This line is visible even beneath the BCP clusters. Upon closer examination, the bright line can be identified as an ultrafine BCP line deposited along with the surface scratch of the quartz. Fig. 6d provides a schematic representation of the sharp contrast observed, which is attributed to the increased thickness of the deposited sample. This distance increase results in a highly sensitive change of the PiDF between the metallic tip and quartz. The demonstration of this phenomenon implies that the present technique of substrate-enhanced nano-IR contrast imaging at tip-induced near-field resonance can be a promising method for subsurface imaging of ultrafine structures.

## CONCLUSION

We have demonstrated a substrate-enhanced nano-IR contrast imaging technique by implementing the strongly enhanced PiDF near the phonon polaritonic resonance. The key idea in this work is to enhance the dipole force near phonon polaritonic resonance via limiting the thermal expansion by choosing a substrate material with a low thermal property such as low thermal expansion coefficient, absorption coefficient or thermal diffusivity. Our approach can be utilized to diagnose comprehensive morphological information of ultrathin structures, including subsurface, which is promising for the non-invasive diagnosis of nanoscale devices by phonon polaritonic materials.

It is worth mentioning that, unless the substrate's thermal properties are negligible, PiTF may still be comparable to PiDF near the IDR [41]. In ref. [41], the authors claim that the surface plasmon polariton (SPP) of the graphene monolayer is mediated by the thermal expansion of thermally prepared SiO_2_/Si substrate. Since the thermally prepared SiO_2_ film can have a larger thermal expansion coefficient than quartz, the PiTF of SiO_2_ substrate can mediate the SPP via thermal expansion. The tapping mode PTIR spectrum on pure quartz is slightly deviated from the previously reported s-SNOM amplitude spectrum [40,42,43], which has a peak near Si–O–Si vibrational absorption as well as the image dipole near-field resonance. However, the PiDF is also expected to be at maximum near the resonance. Indeed, the SPP fringe pattern of graphene at the resonance is inverted and similar to the s-SNOM amplitude (real part). Note that the fringe pattern depends on lots of sample/substrate conditions such as the wavelength of incident beam, doping level and edge conditions, so that it would be better to compare the spectrum with s-SNOM rather than measuring the fringe pattern.

In sum, we propose a method to amplify the sensitivity and contrast of photoinduced force responses in transmission images by implementing PiDF detection on phonon polaritonic substrates, enabling precise differentiation of surface and subsurface distributions in ultrathin samples. As a validation, we apply this method to visualize thin COFs layers and subsurface defects under BCP films. By selecting suitable IR materials that exhibit phonon polaritons/Reststrahlen bands (Table S3), users can achieve high-resolution nanoimaging of specific crystals, polymer molecules and biomolecules (for example, DNA origami structures, shown in Fig. S8) with known vibrational mode frequencies. Additionally, an AFM tip crafted from phononic materials can enhance imaging capabilities, potentially surpassing substrate limitations (Fig. S9).

## METHODS

The detailed experimental procedures can be obtained in the supplementary data.

## Supplementary Material

nwae101_Supplemental_File
