# Supplementary material for: Surface-phonon-polariton-enhanced photoinduced dipole force for nanoscale infrared imaging
Source: Natl Sci Rev. 2024 Mar 18;11(5):nwae101. doi: 10.1093/nsr/nwae101 (PMC11065349; doi:10.1093/nsr/nwae101)
Supplement: nwae101_Supplemental_File [file nwae101_supplemental_file.docx]

**Surface Phonon Polariton Enhanced Photoinduced Dipole Force for Nanoscale Infrared Imaging**

Jian Li,^1†^ Junghoon Jahng,^2†^ Xuezhi Ma,^3^ Jing Liang,^1^ Xue Zhang,^1^ Qianhao Min,^1^ Xiao-Liang Wang,^1^ Shuangjun Chen,^4^ Eun Seong Lee,^2^ Xing-Hua Xia^1*^

^1^ State Key Lab of Analytical Chemistry for Life Science, School of Chemistry and Chemical Engineering, Nanjing University, Nanjing 210023, China

^2^ Hyperspectral Nano-imaging Team, Korea Research Institute of Standards and Science, Daejeon 34113, Republic of Korea

^3^ Institute of Materials Research and Engineering, Agency for Science, Technology and Research, 2 Fusionopolis Way, Innovis, #08-03, Singapore 138634 Singapore

^4^ College of Materials Science and Engineering, Nanjing Tech University, Nanjing, Jiangsu 210009, P. R. China

**Table of contents**

**S1. Experimental section**

**S2. Operation principle of heterodyne mode IR-PiFM**

**S3. Calculation of the tip-enhanced and bulk thermal expansions by implementing finite dipole method (FDM)**

**S4. Modeling of the photo-induced dipole force on layered system by implementing finite dipole method (FDM)**

**S5. Permittivity of PDMS**

**S6. Validation of the analytically calculated E-field with numerical method**

**S7.Comparison of FTIR spectrum and PiF spectrum of COFs**

**S8.Thickness dependent PiF evolution of COFs on quartz**

**S9. Nano-IR contrast imaging of DNA origami on quartz.**

**S10. Summary of reststrahlen band for some phononic substrates.**

**S11. Numerical (COMSOL) calculation of E field induced by tip made of quartz.**

**S1. Experimental Section**

**Material**

Quartz was bought from Suzhou Crystal Silicon Electronic&Technology Co., Ltd (China). AFM tip was purchased from Molecular Vista, Inc. (San Jose, USA). Sylgard184 was bought from Sigma-Aldrich (USA). For COFs synthesis, 4',4''',4''''',4'''''''-(1,2-ethenediylidene)tetrakis[1,1'-biphenyl]-4-carboxaldehyde (ETBC, 98%) was received from Jilin EXTENSION Technology Co., Ltd. 4,4',4'',4'''-(ethene-1,1,2,2-tetrayl)-tetraaniline (ETTA, 97%) were received from Shanghai Tensus Biotech Co., Ltd. o-dichlorobenzene (o-DCB, AR) and n-butanol (AR) were bought from Shanghai Tingxin Chemical Reagent Co., Ltd. Acetic acid (AR) was bought from Sinopharm Chemical Reagent Co., Ltd. Tetrahydrofuran (THF, AR) was purchased from Nanjing Chemical Reagent Co., Ltd..

Asymmetric PS-b-PMMA block copolymers were purchased from Polymer Source with the number-average molecular weight (Mn) 37k g/mol (PS block 21k and PMMA block 10k) and polydispersity index (Mw/Mn) 1.08.

Other reagents and chemicals were of analytical grade. All reagents were used as received without further purification. All solutions were prepared with Milli-Q water from a Millipore system.

**Instruments**

Atomic force microscopic (AFM) and photoinduced force microscopic (PiFM) images were recorded on a VistaScope system (Molecular Vista, USA). Fourier transform infrared (FTIR) spectra were collected on a Nicolet IS50 (Nicolet, USA).

**Preparation of PDMS droplet**

For PDMS droplet preparation, a 10 μL of Sylgard184 was dispersed on top of quartz and mica. After the droplet structures were formed, they were heated at 80 ℃ until curing.

**Preparation of COFs nanostructures**

Typically, 44.9 mg of ETBC, 63.0 mg of ETTA, 0.4 mL of o-DCB/n-butanol (7:3, *v*/*v*) and 0.05 mL 12 M acetic acid were charged into a 2 mL Pyrex tube. The mixture was degassed by three freeze-pump-thaw cycles, followed by flame-sealing under vacuum. Then the pyrex tube was heated at 120 °C for 5 days. After cooling, the mixture was filtered and washed with anhydrous THF several times to remove unreacted monomers, the catalyst, and the solvent. The solid was further purified by Soxhlet extraction using anhydrous THF for 1 day and dried under supercritical CO_2_ flow to obtained a yellow powder.

**Preparation of PS-b-PMMA copolymer films**

To prepare the PS-b-PMMA copolymer films, a 0.5 wt% PS block 21k and PMMA block 10k solution in toluene were spin-coated onto quartz substrates and then annealed at 150 °C in a vacuum for 2 h.

**Measurements of PiFM**

PiFM measurements were taken on a VistaScope microscope that is coupled to a QCL laser system from Block Engineering with a wavenumber resolution of 1 cm^-1^ and a tuning range from 770 to 1885 cm^-1^. The IR beam was focused on the sample with an angle of 30 degrees using a parabolic mirror and had a pulse duration of 30-40 ns. The set point was set as 70 %-90 %. The microscope was operated in a dynamic mode with NCH-Au 300 kHz non-contact cantilevers. The measurements were carried out in an atmosphere environment. The collection time for each spectrum was around 1-3 s, and the time per image was about 10 min with 0.5 line/s speed at a 256×256 resolution.

**E field simulation**

The simulation was performed using the finite element method on commercial software (Comsol-Multiphysics® version 6.0). A scattering field was used to simulate the field distribution. The incident beam was set as p-polarized with 45 degrees angle. The tip was set as a hemisphere with a 30 nm radius head connected to a cone with a 50 nm end radius and 200 nm height. The permittivity of different materials is adapted from literature as indicated in the main text.

**S2. Operation principle of heterodyne mode IR-PiFM**

As diagramed in Figure 3a, for PiFM measurement, the *p*-polarized pulsed quantum cascade laser (QCL) is focused at the tip end of an Au-coated cantilever through an integrated off-axis parabolic mirror. Multifrequency tapping mode AFM operation is implemented; The incident light is modulated by the PiFM heterodyne mode, which is defined as the sum or difference (f_m_=f_2_±f_1_) between the first (f_1_) and second (f_2_) eigenmodes of the cantilever; f_1_ is used to detect the PiFM signal, while f_2_ is used to detect the topography. This method helps to measure the gradient of photoinduced force, which can extract tip-sample distance-dependent response by reducing some constant background force.

**S3. Calculation of the tip-enhanced and bulk thermal expansions by implementing the finite dipole method (FDM)**

The tip-enhanced and bulk thermal expansions can be calculated by implementing the finite dipole model to the three-layered materials[1]. For the bulk thermal expansion, it follows Beer’s law (Schemed in Figure S1). For the tip-enhanced absorption, the E-field inside the sample should be calculated by differentiating the potentials of Q_0_ and Q_1_ (Schemed in Figure S3). The bulk and tip-enhanced thermal expansions are given as:

${\Delta L}_{b}(z)=\frac{\sigma l_{z}\tau_{\mathrm{th}}}{\rho CV_{\mathrm{heat}}}\int a_{\mathrm{abs}}\frac{1}{2}{c\epsilon}_{0}|E_{f}|^{2}dV_{\mathrm{abs}}$ (S1)

${\Delta L}_{t}(z)=\frac{\sigma l_{z}\tau_{\mathrm{th}}}{\rho CV_{\mathrm{heat}}}\int a_{\mathrm{abs}}\frac{1}{2}{c\epsilon}_{0}|E_{n}|^{2}dV_{\mathrm{abs}}$, (S2)

where $\tau_{\mathrm{th}}=\tau_{\mathrm{rel}}(1-e^{{-t_{p}}/{\tau_{\mathrm{rel}}}})$, $\tau_{\mathrm{rel}}=\frac{4}{\pi^{2}}\frac{\rho C}{\kappa_{\mathrm{eff}}}l_{z}^{2}$, $a_{\mathrm{abs}}=\frac{4\pi}{\lambda}\mathrm{Im}[n]$, $|E_{f}|^{2}\approx(1-R)^{2}|E_{0}|^{2}=\frac{9Re[n]}{{(Re[n]}^{2}+2)^{2}}|E_{0}|^{2}$ and $|E_{n}|^{2}=|\frac{\partial U_{n}}{\partial z'}|^{2}$. The typical value of the parameters are When we define the effective absorption coefficient as ${a^{'}}_{abs}\left( \lambda\right)\approx a_{\mathrm{abs}}\frac{9Re[n]}{{(Re[n]}^{2}+2)^{2}}=\frac{4\pi}{\lambda}\frac{9Re[n]\mathrm{Im}[n]}{{(Re[n]}^{2}+{2)}^{2}}$,[2] by implementing the complex refractive index from the complex permittivity of quartz [3] with the relation of $\varepsilon'={\mathrm{Re}[n]}^{2}-{\mathrm{Im}[n]}^{2}$ and $\varepsilon\text{''}=2\mathrm{Re}[n]\mathrm{Im}[n]$, the effective absorption coefficient and the total thermal expansion are calculated and the results are shown in Figure S1c and d.

The physical meaning of $\tau_{\mathrm{th}}$, $\tau_{\mathrm{rel}}$ and $l_{z}$ are the effective thermalization time, i.e. the time taken to reach equilibrium between heating and cooling, relaxation (cooling) time and effective heating length. The $l_{z}$ can be regarded as the total sample thickness for a thin sample. The calculated $\tau_{\mathrm{rel}}$ and $\tau_{\mathrm{th}}$ of PDMS is plotted as below. $\tau_{\mathrm{th}}$ shows saturation behavior while the $\tau_{\mathrm{rel}}$ shows proportional behavior to the heated length. Since most polymers shows similar thermal conductivity, density and heat capacity, $\tau_{\mathrm{rel}}$ and $\tau_{\mathrm{th}}$ of polymers show similar orders of magnitude with respect to the sample thickness.


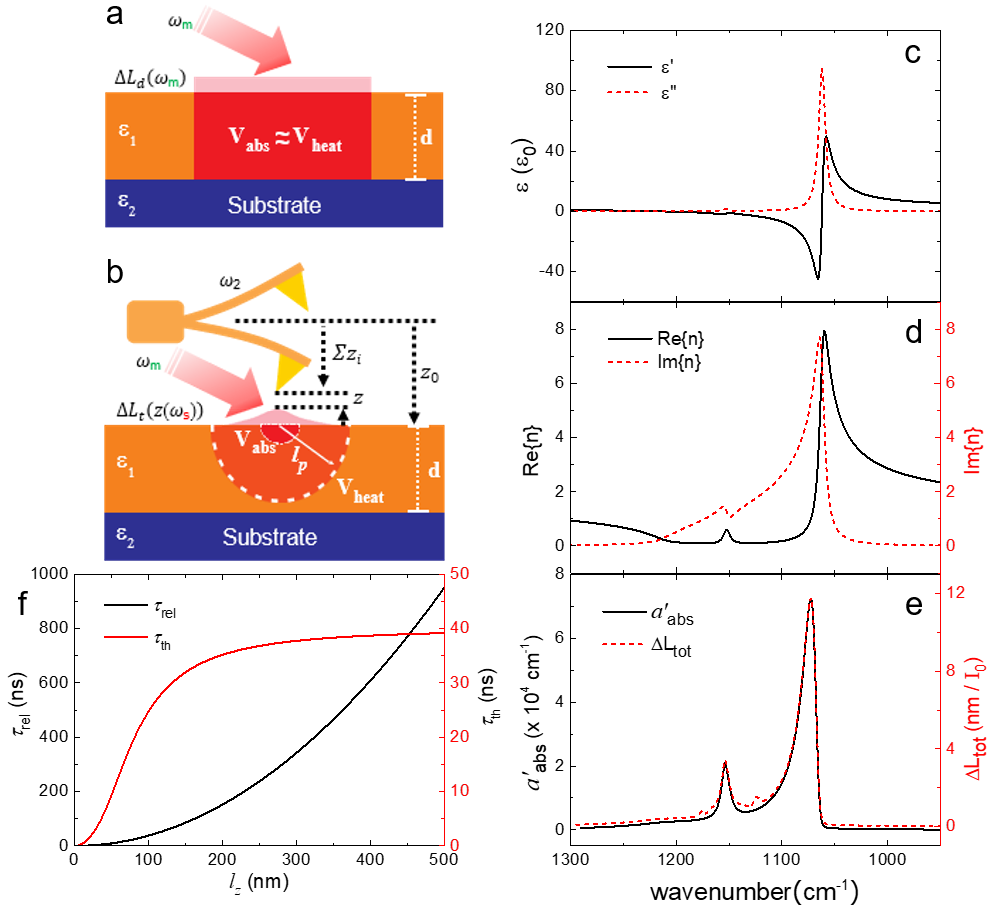


**Figure S1. Schematics of thermal expansions and corresponding calculations.** (a) Bulk thermal expansion of a quartz substrate based on the direct incident field and (b) tip-enhanced thermal expansion of quartz based on the near field. (c) Complex permittivity of quartz from library data[3]. To fit our quartz data, they are red-shifted by 10 cm^-1^. (d) Calculated complex refractive index from (c) by using the relation of $\varepsilon'={\mathrm{Re}[n]}^{2}-{\mathrm{Im}[n]}^{2}$ and $\varepsilon\text{''}=2\mathrm{Re}[n]\mathrm{Im}[n]$. (e) Calculated effective absorption coefficient and the total thermal expansion. (f) Calculated relaxation time ($\tau_{\mathrm{rel}}$) and effective thermalization time ($\tau_{\mathrm{th}}$) of PDMS with respect to the heated length ($l_{z}$).


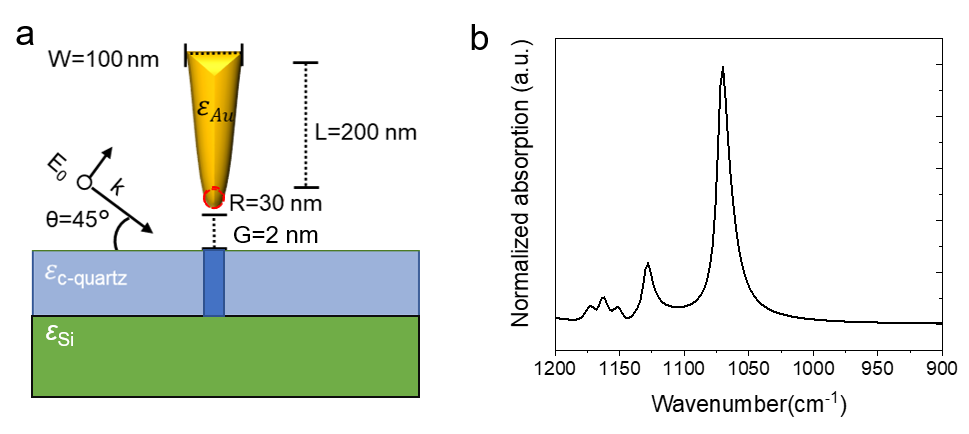


**Figure S2.** Numerical (COMSOL) calculation of quartz absorption below the illuminated Au tip. (a) Sketch of COMSOL calculation geometry. Thickness of quartz:1 μm. (b) Simulated tip induced absorption of the quartz below tip with different wavenumber illumination.

In addition, a numerical simulation was further carried out to compare the tip enhanced absorption and bulk absorption of quartz (Figure S2), showing that for a bulk quartz, the tip induced absorption at 1130 cm^-1^ is much smaller as compared with bulk absorption at 1070 cm^-1^. Thus, the PiTF at 1130 cm^-1^ should be weaker than at 1070 cm^-1^. The stronger PiF peak observed at 1130 cm^-1^ than at 1070 cm^-1^ in our experiment cannot be interpreted as a phonon enhanced PiTF.

**S4. Modeling of the photo-induced dipole force on the layered system by implementing the finite dipole method (FDM)**

When a sharp metal tip is illuminated, the lightning-rod effect confines E-fields to the end of the tip. This effect can be modeled with the finite dipole method with respect to the geometry of the tip[1]. The schematic diagram in the tip-sample geometry is illustrated in Figure S3. The tip is modeled as an ellipsoid of length *2L* and the tip end is described as a sphere of radius R, which is inscribed in the ellipsoid. The electric field from the tip without the sample can be successfully mimicked by a charge (Q_0_) which lies in the center of the inscribed sphere[4]. The charge Q_0_ is described as:

$Q_{0}=4\pi\varepsilon_{0}R^{2}\frac{\frac{2F(L)}{L(R)}+\mathrm{Log}[\frac{L-F}{L+F}]}{\frac{2F(L-\varepsilon_{t}R)}{La(\varepsilon_{t}-1)}-\mathrm{Log}[\frac{L-F}{L+F}]}E_{0}$ (S3)

where *ε_0_* is the permittivity of the vacuum, *F* is the focal length given as $F=L\sqrt{1-\frac{R}{L}}$, *z* is the distance from the tip end to the sample surface and the *ε*_t_ is the permittivity of the tip.


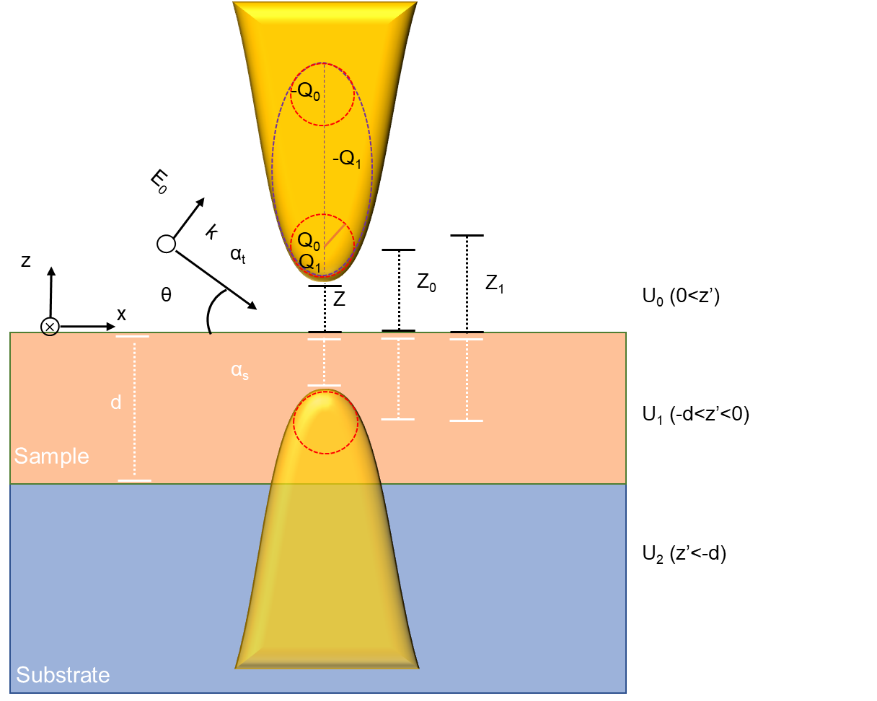


**Figure S3. Schematic diagram of image charges and related tip-enhanced thermal expansion based on ellipsoidal finite dipole model.** The tip is modeled as an ellipsoid with a length of *2L*. The plane wave light is illuminated to the sample with an angle of *θ*. *z* is the gap distance from the tip to the sample surface and *d* is the thickness of the sample.

On a planar surface, the field enhancement near the tip-end is boosted by the multiple-scattering process between the tip and the substrate[4, 5]. Hauer *et al*. [6] successfully modeled the multiple-scattering effect on the layered system. In this model the two charges are induced on the tip: one is the initial charge (Q_0_) due to the incident beam E_0_, which is positioned at the center of the inscribed sphere *R*. The other one is the induced charge (Q_1_) due to the potential response of the sample. The potential response *U* of the sample to the potential of a charge Q_0_ at the distance *z_0_* = *R+z* above a flat layered sample [6].

The electric neutrality of an isolated spheroid requires the existence of a charge -Q_i_ somewhere on the spheroid. The dipole moment *p*_i_ can be derived by considering that the charge -Q_i_ must not break the equipotential property of the spheroid. For this reason, we can assign a dipole moment *p*_i_ to the total charge distribution oscillating with the driving field frequency. For symmetry reasons we thus can locate the total amount of -Q_i_ into the spheroid center yielding *p*_i_ = Q_i_ *L*. Subsequently, the initial dipole moment of the tip is given as *p*_0_ = 2Q_0_*L* = α_t_E_0_ and the dipole moment arising from the near-field interaction is given as *p*_1_ = ηQ_0_*L* = ηα_t_E_0_/2 where η is near-field enhancement factor, given as $\eta=\frac{Q_{i}}{Q_{0}}$.

The total force between the tip and substrate is possible to implement the above-mentioned static charge approach. The electrostatic force (monopole) is directly calculated by using the Coulombic force between the charges on the tip and the sample, which is given as:

$F_{\mathrm{monopole}}=-\frac{1}{4{\pi\varepsilon}_{0}}\mathrm{Re}\{\frac{\beta_{X_{0}}|Q_{0}|^{2}}{{(z_{0}+X_{0})}^{2}}+\frac{{(Q_{0})}^{*}\beta_{X_{1}}Q_{1}}{{(z_{0}+X_{1})}^{2}}+\frac{{(Q_{1})}^{*}\beta_{X_{0}}Q_{0}}{{(z_{1}+X_{0})}^{2}}+\frac{\beta_{X_{1}}|Q_{1}|^{2}}{{(z_{1}+X_{1})}^{2}}\}$ (S4).

where $X_{0}\approx z+R$ and $X_{1}\approx z+R/2$. $\beta_{X_{i}}$ is the near-field reflection factor for multilayer, givne as $\beta_{X_{i}}=\frac{U_{i}^{2}}{U_{i}^{'}}|_{z=0}$. For the single layer isotropic material, the equation is rewritten as:

$$F_{\mathrm{monopole}}=-\frac{1}{4{\pi\varepsilon}_{0}}\mathrm{Re}\{\frac{\beta}{{(2H+2R)}^{2}}+\frac{\beta|\eta|^{2}}{{(2H+R)}^{2}}+\frac{\eta^{*}\beta}{{(2H+1.5R)}^{2}}+\frac{\beta\eta}{{(2H+1.5R)}^{2}}\}{{|Q}_{0}|}^{2}$$

where $\eta=\frac{\beta(g-\frac{R+H}{L})\ln\frac{4L}{4H+3R}}{\ln\frac{4L}{R}-\beta(g-\frac{3R+4H}{4L})\ln\frac{2L}{2H+R}}$ for an isotropic material. The real part of *β* reaches its maximum at ε’=-1 without considering the imaginary part of permittivity (ε’’=0). However, if we consider the imaginary part of permittivity, the resonance of *β* changes. Given that the complex variable of *η* includes complex *β* in both the numerator and denominator of the force equation, involving specific geometry parameters such as *R, H, L*, and *g*, the spectral shift can occur with respect to tip-sample geometry. Thus, the maximum dipole force (F_dip_) arises at 1125 cm-1 (*ε*’=-3.07 and *ε*’’=0.39) as shown in the image below with blue solid line. The spectral dependence of *β,* *η* and F_dip_ are also depicted in the below image. The incident electric field *E*_0_ can be considered as the peak field for the pulsed beam, which can be normalized by the incident power as $E_{0}=\sqrt{\frac{2}{c\epsilon_{0}}\frac{I_{0}}{f\tau_{p}}}$ where *I_0_* = 5 mW, repetition rate of *f* = 1.6 MHz and pulse width of *τ_p_* = 40 ns. The simulation parameters are *R* = 30 nm, *L* = 300 nm, *t*_p_ = 40 ns, *z* = 1 nm, and *θ =* 40 degrees. For better comprehension, we have summarized the estimated values of parameters used in our experiments and calculations in Table S1.

**Table S1.** Estimated values of parameters used in experiments and calculations

| Focal spot size | 2λ,10-25 μm |
| --- | --- |
| Incident laser power | ~5 mW |
| Repetition rate | ~ 2MHz |
| Pulse width | 40 ns |
| Duty cycle | ~ 0.08 |
| Incident E_0_ | ~ 10^5^ V/m |

**
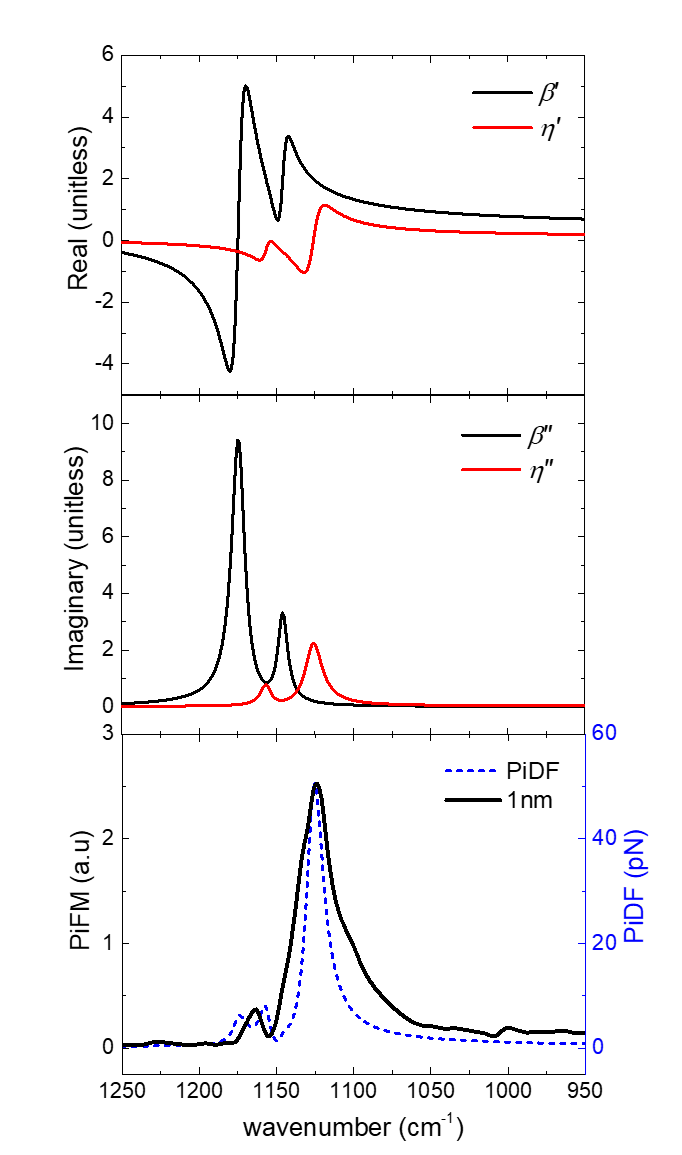
**

**Figure S4. Calculated complex near-field reflection factor *β*, near-field enhancement factor *η* and the photoinduced dipole force at H = 1 nm and measured PiFM spectrum.**

**S5. Permittivity of PDMS**


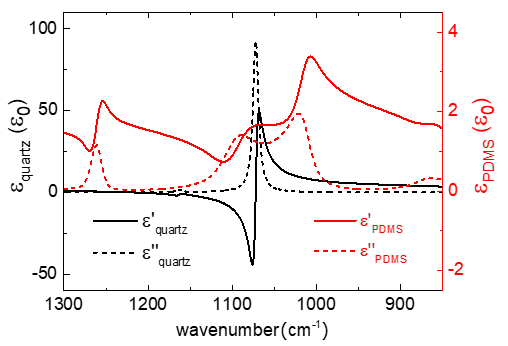


**Figure S5**. The relative permittivity of PDMS[7] (red) and quartz[3] (black) with respect to the wavenumber from the library data. The permittivity of quartz is red-shifted by 10 cm^-1^ from Ref. [3].

**S6. Validation of the analytically calculated E-field with numerical method**

Numerical calculation of E-field was performed using the finite element method on commercial software (Comsol-Multiphysics® version 6.0). A scattering field was used to simulate the field distribution. The incident beam was set as *p*-polarized with 45 degrees angle. The wavenumber was set as 1130 cm^-1^ and 1260 cm^-1^. The tip, which is modeled as a hemisphere with a 30 nm radius head connected to a cone with 50 nm end radius and 200 nm height, is located on the PDMS film on the quartz substrate by the gap distance of 1 nm, as shown in Figure S6a. In Figure S6b, the analytical calculations (solid lines) well correspond to the COMSOL simulation (square dots) at the gap-field resonance between tip and quartz (1130 cm^-1^) as well as the vibrational resonance of PDMS (1260 cm^-1^). The scale of the PiFM signal at 1260 cm^-1^ is inverted to directly compare to the one at 1130 cm^-1^. The field at the gap-field resonance is more surface sensitive than the field at the vibrational resonance of the molecule.


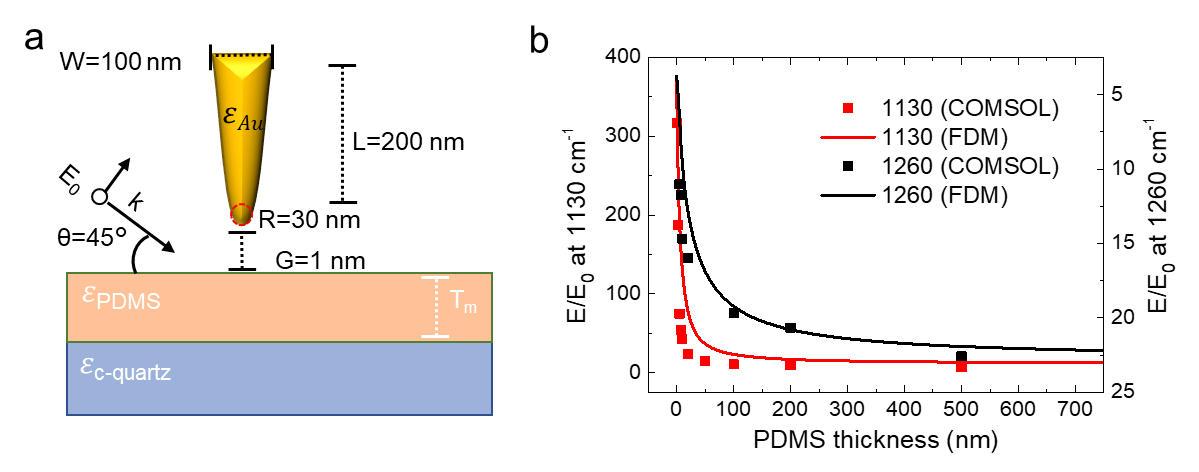


**Figure S6. Comparison between analytical (FDM) and numerical (COMSOL) calculation. (**a) Sketch of COMSOL calculation geometry. (b) Comparison between FDM (solid lines) and COMSOL (dots) at 1130 cm^-1^ and 1260 cm^-1^, respectively. The scale of the PiFM signal at 1260 cm^-1^ is inverted to compare with the one at 1130 cm^-1^.

**S7.Comparison of FTIR spectrum and PiF spectrum of COFs**

**Figure S7. Comparison of FTIR spectrum (red curve) and PiF spectrum of COFs on quartz (black curve).**

**S8.Thickness dependent PiF evolution of COFs on quartz**

**
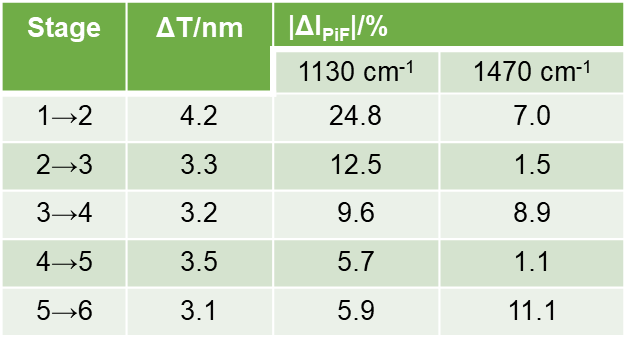
**

**TableS2. Thickness dependent PiF evolution of COFs on quartz.**

**S9. Nano-IR contrast imaging of DNA origami on quartz.**

**
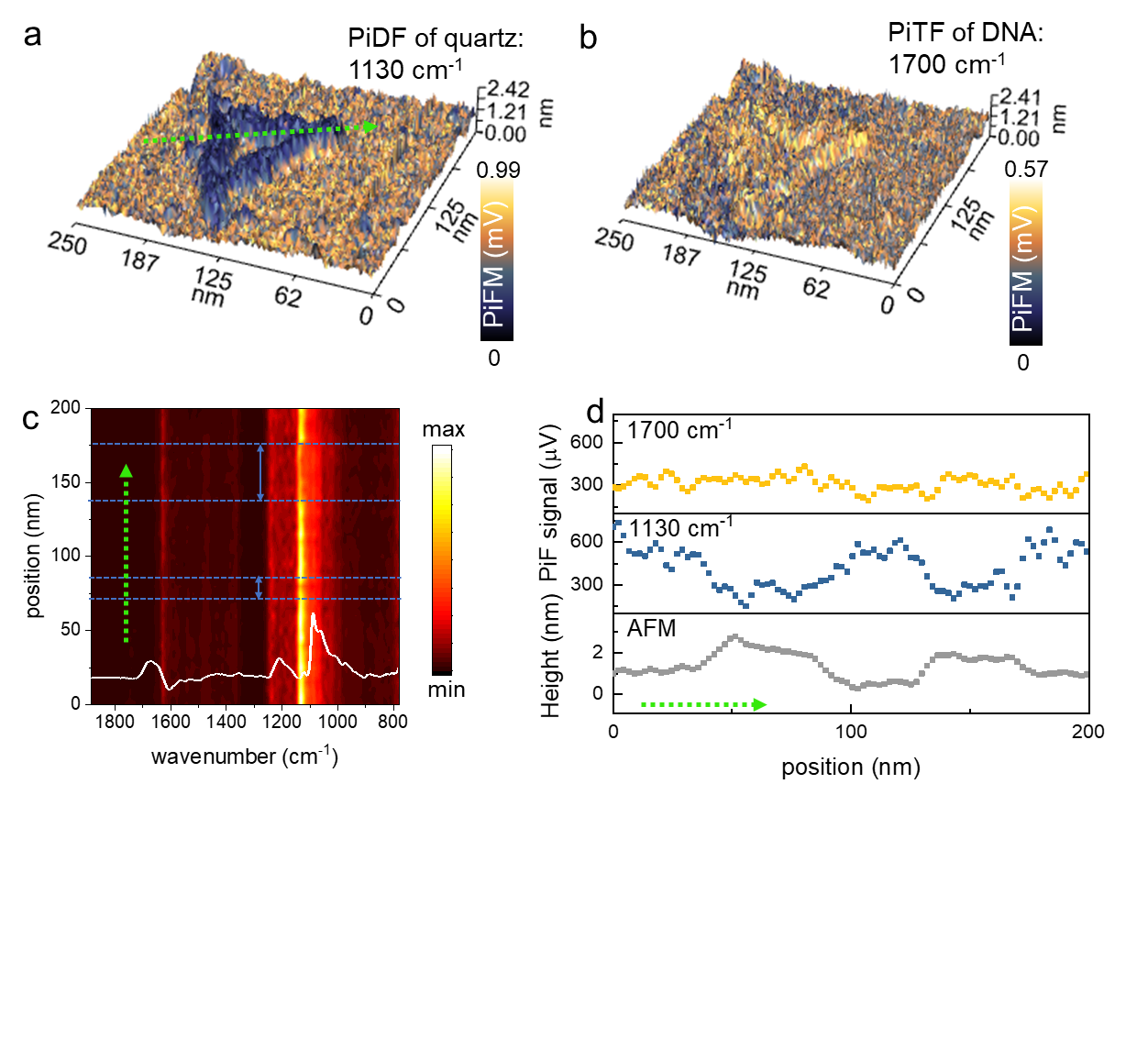
**

**Fig. S8. Nano-IR contrast imaging of DNA origami on quartz.** (a-b) Merged AFM and PiFM images of DNA origami on a quartz surface at (a) 1130 cm^-1^ and (b) 1700 cm^-1^, respectively. (c) Corresponding spatio-spectral transection along the green dashed arrows in (a). Spectra were obtained in 3 s with 1 cm^−1^ spectral resolution, with 5 nm separation. The white curve represents the FTIR spectrum of a random DNA sequence. Spectra between the blue dashed lines were recorded on top of DNA origami and the rest were observed on quartz. (d) Signals along the green arrow of topography and PiFM images in (a-b), respectively.

DNA origami, a kind of well-organized nanostructures programmed via base-pairing, as representative of thin samples was investigated. The AFM image (Fig. S8a and b) of DNA origami exhibits a hollow equilateral triangle shape with a lateral side length of approximately 130 nm, a medial side length of around 43 nm, and a height of less than 2 nm. The FTIR spectrum of a self-assembled monolayer of randomly selected oligonucleotide strand displays three characteristic peaks at 1680 cm^-1^, 1205 cm^-1,^ and 1086 cm^-1^ (white curve in Fig. S8c), which are assigned to the double bonds C=O and C=C stretching, asymmetric stretching vibration of P−O bonds, and symmetric vibration of P=O bonds from phosphate backbone groups, respectively. However, these peak features are not observed in the spatio-spectral transection of PiF spectra recorded on DNA origami (Fig. S8c). As compared with the PiF spectra on quartz, only a strong but reduced peak centered at around 1130 cm^-1^ is observed, which can be attributed to the PiDF signal from the quartz.

Nano-IR contrast images of DNA origami were captured at two representative wavenumbers: at PiDF of quartz of 1130 cm^-1^ (Fig. S8a) and at PiTF of DNA origami of 1700 cm^-1^ (Fig. S8b). The hollow triangle shape of DNA origami can be easily distinguished from the PiDF signal of quartz with a strong negative contrast as shown by the merged PiFM images with AFM images. At the same time, the PiFM images with PiTF of DNA origami display negligible contrasts with an overall signal of 300 μV. These features are noticeable in the extraction of AFM and PiFM signals along the same line profile (Fig. S8d), revealing the DNA thickness-induced PiF signal evolution. Despite the applied laser power density being 10 times smaller for imaging with a signal of 1130 cm^-1^ compared to the signal at 1700 cm^-1^, we observed the three times improved signal with better contrast.

**S10. Summary of reststrahlen band for some phononic substrates.**

| **Substrates** | **Direction** | **TO(cm^-1^)** | **LO(cm^-1^)** | **Reference** |
| --- | --- | --- | --- | --- |
| α-MoO_3_ | <001> (Y) | 544.6 | 850.1 | [8] |
|  | <100> (X) | 821.4 | 963 | [8] |
|  | <010> (Z) | 998.7 | 999.2 | [8] |
| α-V_2_O_5_ | <001> (Y) | 506 | 842 | [9] |
|  | <100> (X) | 765 | 952 | [9] |
|  | <010> (Z) | 976 | 1037 | [9] |
| h-BN | in-plane | 1369.8 | 1612.9 | [10] |
|  | out-of-plane | 757.6 | 826.4 | [10] |
| 6H-SiC | Bulk | 793.9 | 970.1 | [11] |
| c-AlN | in-plane | 614 | 893 | [12] |
|  | out-of-plane | 673 | 916 | [12] |
| c-GaN | in-plane | 533 | 735 | [12] |
|  | out-of-plane | 561 | 743 | [12] |
| α-Quartz | ⊥ to c-axis | 1071.8 | 1215.1 | [13] |
|  |  | 450 | 522.5 | [13] |
|  |  | 394 | 420.7 | [13] |
|  | \|\| to c-axis | 1079.9 | 1222.5 | [13] |
|  |  | 495 | 559.3 | [13] |
|  |  | 364 | 412.7 | [13] |

**Table S3.** Summary of reststrahlen band for some phononic substrates.

**S11. Numerical (COMSOL) calculation of E field induced by tip made of quartz.**

**
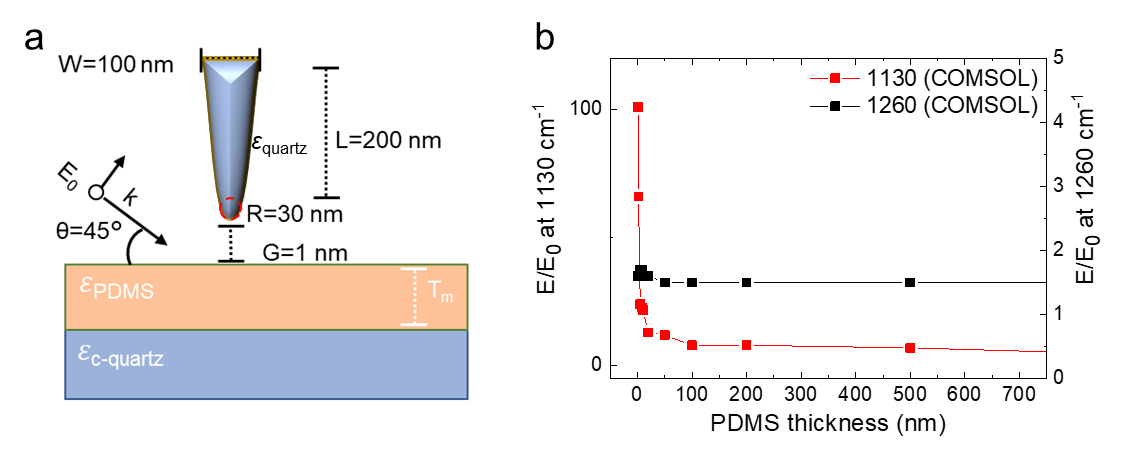
**

**Figure S9. Numerical (COMSOL) calculation of E field induced by tip made of quartz. (**a) Sketch of COMSOL calculation geometry. (b) Comparison between E field induced by tip made of quartz at 1130 cm^-1^ and 1260 cm^-1^, respectively. The scale of the PiFM signal at 1260 cm^-1^ is inverted to compare with the one at 1130 cm^-1^.

It can be seen that the tip made of phononic materials can also create gap modes for enhancing the E field (Figure S7), which is benefitial for nanoimaging.

**References:**

1. Jahng J, Potma EO, Lee ES; Tip-enhanced thermal expansion force for nanoscale chemical imaging and spectroscopy in photoinduced force microscopy. *Anal Chem* 2018;**90**:11054-61.

2. Lin Y-T, He H, Kaya H, et al.; Photothermal atomic force microscopy coupled with infrared spectroscopy (AFM-IR) analysis of high extinction coefficient materials: a case study with silica and silicate glasses. *Anal Chem* 2022;**94**:5231-9.

3. Amarie S, Keilmann F; Broadband-infrared assessment of phonon resonance in scattering-type near-field microscopy. *Phys Rev B* 2011;**83**:045404.

4. Cvitkovic A, Ocelic N, Hillenbrand R; Analytical model for quantitative prediction of material contrasts in scattering-type near-field optical microscopy. *Opt Exp* 2007;**15**:8550-65.

5. Zhang C, Chen B-Q, Li Z-Y; Optical origin of subnanometer resolution in tip-enhanced raman mapping. *J Phys Chem C* 2015;**119**:11858-71.

6. Hauer B, Engelhardt AP, Taubner T; Quasi-analytical model for scattering infrared near-field microscopy on layered systems. *Opt Exp* 2012;**20**:13173-88.

7. Singleton EB, Shirkey CT; Optical constants in the IR from thin film interference and reflectance: the reststrahlen region of muscovite mica. *Appl Opt* 1983;**22**:185-9.

8. Alvarez-Perez G, Folland TG, Errea I, et al.; Infrared permittivity of the biaxial van der Waals semiconductor alpha-MoO(3) from near- and far-field correlative studies. *Adv Mater* 2020;**32**:e1908176.

9. Taboada-Gutierrez J, Alvarez-Perez G, Duan J, et al.; Broad spectral tuning of ultra-low-loss polaritons in a van der Waals crystal by intercalation. *Nat Mater* 2020;**19**:964-8.

10. Caldwell JD, Aharonovich I, Cassabois G, et al.; Photonics with hexagonal boron nitride. *Nat Rev Mater* 2019;**4**:552-67.

11. MacMillan MF, Devaty RP, Choyke WJ, et al.; Infrared reflectance of thick p-type porous SiC layers. *J Appl Phys* 1996;**80**:2412-9.

12. Ratchford DC, Winta CJ, Chatzakis I, et al.; Controlling the infrared dielectric function through atomic-scale heterostructures. *ACS Nano* 2019;**13**:6730-41.

13. Foteinopoulou S, Devarapu GCR, Subramania GS, et al.; Phonon-polaritonics: enabling powerful capabilities for infrared photonics. *Nanophotonics* 2019;**8**:2129-75.
